# Receptor conversion and survival in breast cancer liver metastases

**DOI:** 10.1186/s13058-023-01706-4

**Published:** 2023-09-13

**Authors:** Marcus Sundén, Sofia Norgren, Robert Lundqvist, Anne Andersson, Malin Sund, Oskar Hemmingsson

**Affiliations:** 1 Department of Surgical and Perioperative Sciences/Surgery, Umeå University, 901 85 Umeå, Sweden; 2 Department of Public Health and Clinical Medicine, Umeå University, Umeå, Sweden; 3 Department of Radiation Sciences/Oncology, Umeå University, Umeå, Sweden; 4 Department of Surgery, Helsinki University Hospital, University of Helsinki, Helsinki, Finland; 5 Wallenberg Centre for Molecular Medicine, Umeå University, Umeå, Sweden

**Keywords:** Breast cancer, Liver metastases, Estrogen receptor, Progesterone receptor, Human epidermal growth factor receptor 2

## Abstract

**Background:**

Breast cancer liver metastases (BCLM) is a common cause of breast cancer-related death. The prognostic and predictive value of receptor expression and St Gallen classification is challenged by receptor status discordance in distant metastases. The aim of this study was to determine the rate of receptor conversion from breast cancer to BCLM and the impact on survival.

**Method:**

Patients registered with BCLM in two Swedish national cancer registers were recruited retrospectively. Data on receptor expression in primary breast cancer and BCLM were collected, as well as information about predictive factors for survival. The rate of receptor and subtype conversion was analyzed. A Cox regression model was used to investigate predictive factors for survival.

**Results:**

A cohort of 132 patients with BCLM was identified. Estrogen receptor (ER), progesterone receptor (PgR) and HER2 converted in 17, 33 and 10%, respectively. PgR was lost in BCLM while 8/10 HER2 conversions went from negative to positive. The BC subtype was re-classified in 21% of the BCLM. Median survival after BCLM was 13 months and HER2 amplification was associated with improved survival (HR 0.28 CI 0.085–0.90). The highest predictive value (Harrell´s C-index) was obtained when including both BC and BCLM status.

**Conclusions:**

Receptor and subtype conversions are common in BCLM, and a liver biopsy is warranted to tailor BCLM treatment. HER2 amplification is associated with improved survival in a BCLM cohort.

## Introduction

Advanced breast cancer has a poor prognosis with a median survival of 2–3 years [1]. Breast cancer liver metastases (BCLM) occur in 20–30% of patients with advanced disease, making the liver the third most common site of distant metastases, after bone and lung [2, 3]. In addition, BCLM is a common cause of cancer-related death whereas bone metastases may respond to palliative treatment for a long time [2]. Local treatment of oligometastatic disease, such as isolated BCLM, has come into focus in recent years and is currently evaluated in a randomized clinical trial [4]. However, chemotherapy and endocrine therapy remain the basis for treatment of disseminated disease.

Breast cancer treatment is based on prognostic and predictive factors such as tumor stage, histological grade, proliferation, the pattern of hormonal receptor expression (ER, estrogen receptor; PgR, progesterone receptor) and HER2 amplification (Human epidermal growth factor receptor 2). According to the St Gallen classification, breast cancers can be classified into five intrinsic subtypes with both prognostic and predictive value [5]. The basis for this classification is expression of hormonal receptors and HER2 gene amplification in combination with tumor grade (NHG, Nottingham Histologic Grade) and proliferation (measured by the marker Ki-67). Previous studies have shown that receptor expression and subtype may change from primary breast cancer to distant metastases with potential implications for choice of therapy [6–11]. Thus, standard of care is a biopsy and a re-classification of the metastases [1, 12]. Regarding BCLM, a biopsy carries a theoretical risk of cancer cell seeding as well as a risk for bleeding and infection [13, 14]. Previous studies on receptor conversion are mostly based on extrahepatic metastases. It is therefore important to specifically study the predictive value of BCLM receptor conversion, especially since local treatment of isolated BCLM is currently under investigation [4, 15].

In this study, patients with BCLM were recruited from two national cancer registers. The aim was to analyze the receptor conversion rate from primary breast cancer (BC) to liver metastases. We also asked how conversion affects survival and determined the predictive value of the subtype in the primary cancer and BCLM, respectively.

## Method

### Patient selection

**Fig. 1:**
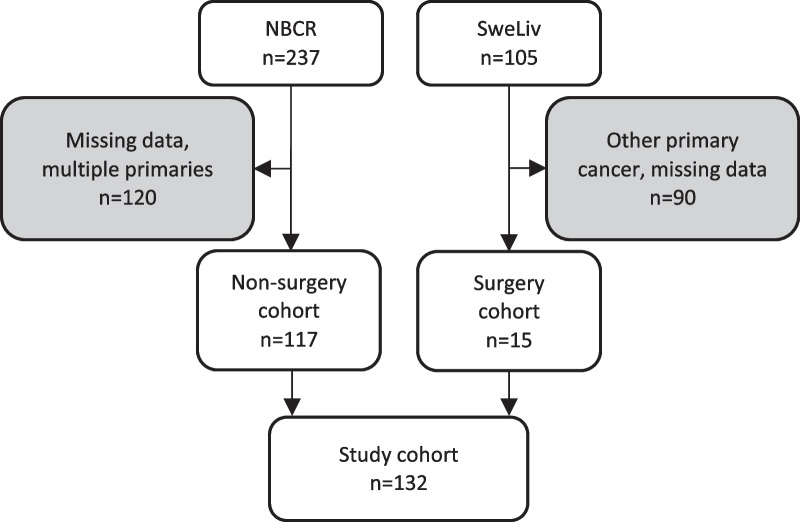
Flow chart for patient inclusion. NBCR, National Breast Cancer Registry; SweLiv, National Register for Cancer in the Liver and Bile ducts

A flowchart of patient selection is shown in Fig. 1. Patients were included from two Swedish national cancer registers, the national register for cancer in the liver and bile ducts (SweLiv) and the national breast cancer register (NBCR). NBCR provided characteristics of the primary tumor, including receptor status. In NBCR, distant metastases are registered on a follow-up form 5 years after breast cancer diagnosis. The SweLiv cohort consists of patients who had surgery for BCLM.

Patients registered in one of these registers between 2009 and 2016 with BCLM and no registered extrahepatic disease were included and followed until 2017–12–31 as in a previous study [15]. Exclusion criteria were unavailable pathological anatomical diagnosis from BC or BCLM and patients with multiple primary breast cancers. To validate register data and add information about BCLM, we studied selected parts of patient medical records. In all participants, pathology reports from BCLM and imaging reports within three months prior to the date of BCLM diagnosis were studied.

### Conversion

Receptor status was considered positive if the pathology report stated so or if the fraction of positive cancer cells was > 10% for ER and > 20% for PgR [16]. HER2 positivity was analyzed with IHC (Immuno Histo Chemistry) and in borderline cases confirmed with FISH-analysis (Fluorescent In Situ Hybridization).

**Fig. 2:**
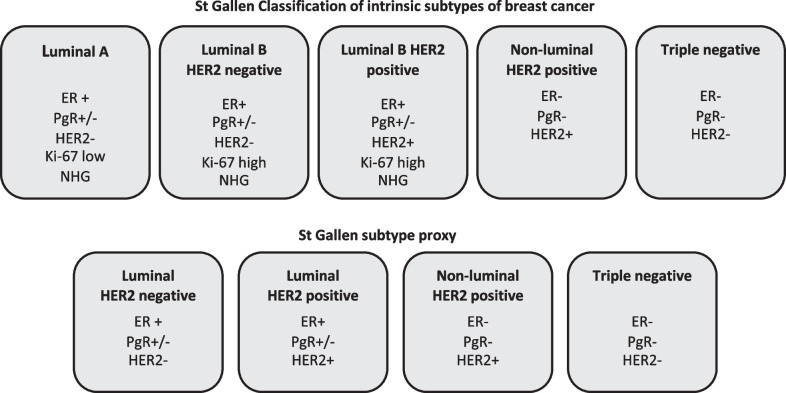
Tumor subtypes. The subtype proxies were used in this study. ER, Estrogen receptor; PgR, Progesterone receptor; HER2, Human Epidermal growth factor receptor 2; Ki-67; marker of proliferation; NHG, Nottingham Histologic Grade

Based on receptor expression, tumors were classified according to the St Gallen classification [5]. Ki-67 and Nottingham histologic grade is used to separate luminal A and B tumors. Ki-67 was not routinely analyzed during the whole study period and histological grade was not reported in the metastases. For the purpose of this study, all luminal HER2-negative tumors were studied as one group. Thus, the four subtypes were luminal HER2 negative, luminal HER2 positive, non-luminal HER2 positive and triple negative breast cancer (TN) (Fig. 2). Comparisons were made between BC and BCLM to analyze the pattern of conversion for receptors and subtypes.

### Predictive factors and survival

Data on the following prognostic and predictive factors were collected; age at time of BC, age at time of BCLM, time from BC to BCLM, receptor expression on cancer cells (ER, PgR, HER2), TNM classification at diagnosis of BC, tumor grade (NHG), vascular ingrowth in BC, number of BCLM and extrahepatic metastases at time of BCLM.

To evaluate the predictive value of receptor expression and subtype in BC compared to BCLM, survival was calculated from date of BCLM diagnosis. In all survival analyses, the cohort from SweLiv was excluded since they had surgery for BCLM and an inherent risk of selection and treatment bias.

### Statistics

Descriptive statistics was used to describe baseline data for the study cohort.

Conversion of receptors and subtypes was analyzed using crosstabs and Chi-2 test.

To analyze predictive factors, a cox regression model was used. All factors with a *p* value < 0.2 in the univariate analysis were used in the multivariable analysis. A *p* value < 0.05 was considered significant. Harrell´s C-index was used to estimate the predictive value of receptor expression in BC compared to BCLM. Kaplan–Meier and log rank test were used to analyze survival. All statistical analyses were made using SPSS Statistics version 26.0, IBM.

## Results

### Patient selection and baseline data

In the NBCR, 237 patients were registered with isolated BCLM. We excluded those where pathological anatomical reports from BC or BCLM were unavailable and patients with multiple primary breast cancers, resulting in 117 inclusions from the NBCR. In addition, 15 patients who underwent liver resection for isolated oligometastases in the liver were included from SweLIV. Thus, the final study cohort consisted of 132 patients (Fig. 1). A review of pathological anatomical reports and imaging reports validated that all had BCLM. The review of radiology reports showed that 53 (40.2%) had extrahepatic metastases (25 bone; 10 lungs; 27 lymph nodes; 11 other). Patient characteristics are shown in Table 1. All patients were females and median age was 60 years (range 27–84) and median time from BC to BCLM was 31 months (range 1–219).

**Table 1:** Patient characteristics and baseline data

	*n* (%)
Cohort	132
Gender (female/male)	132/0 (100/0)
Median Age at BC (years)	60 (27–84)
Tumor size BC	
0	10 (7.9)
1	38 (30.2)
2	62 (49.2)
3	12 (9.5)
4	4 (3.2)
Lgllmet BC	
0	86 (67.7)
1	37 (29.1)
2	2 (1.6)
3	2 (1.6)
Met BC	
No	132 (100.0)
Yes	0 (0.0)
ER BC	
Negative	94 (71.2)
Positive	38 (28.8)
PgR BC	
Negative	57 (43.8)
Positive	73 (56.2)
HER2 BC	
Negative	17 (13.8)
Positive	106 (86.2)
Vascular invasion	
No	33 (32.0)
Yes	70 (68.0)
NHG	
1	4 (3.2)
2	47 (37.9)
3	73 (58.9)
Extrahepatic metastases at time of BCLM	53 (40.2)
Surgery for BCLM	15 (11.4)
Median time BC to BCLM (months)	31 (1–219)
Adjuvant endocrine therapy (BC)	81/116 (69.8)
Adjuvant chemotherapy (BC)	73/117 (62.4)
Adjuvant target therapy (BC)	10/117 (8.5)

BC, primary breast cancer; BCLM, Breast Cancer Liver Metastases; Lgllmet, lymph node metastases; Met BC, distant metastases at time of BC; ER, Estrogen receptor; PgR, Progesterone receptor; HER2, Human Epidermal growth factor receptor 2; NHG, Nottingham Histologic Grade. Extrahepatic metastases, at time of BCLM

The registers lack information on medical treatment for metachronous metastatic disease but data on adjuvant medical treatment for the primary breast cancer indicate that it was administered according to Swedish national guidelines. Treatment included endocrine therapy (81/116; 69.8%), chemotherapy (73/117; 62.4%) and anti-HER2 therapy (10/117; 8.5%). Of those who received chemotherapy, 48/73 (65.8%) were given anthracycline-based therapy. Ten of 13 HER2 positive patients received trastuzumab.

### ER, PgR and HER2 conversion

**Fig. 3:**
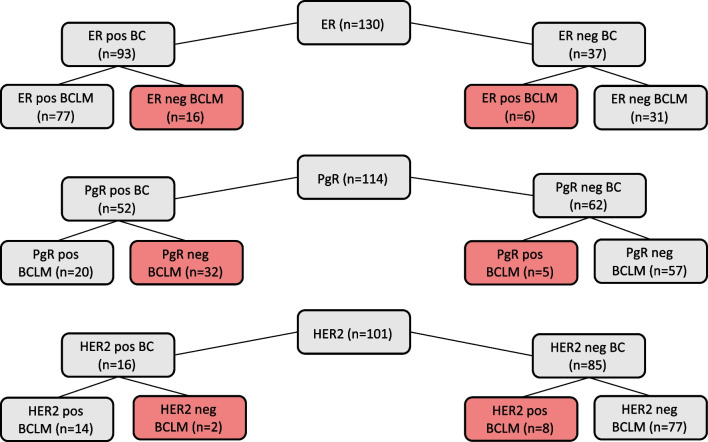
Receptor conversion. ER, Estrogen receptor; PgR, Progesterone receptor; HER2, Human Epidermal growth factor receptor 2; BC, primary breast cancer; BCLM, Breast Cancer Liver Metastases

The expression of hormonal receptors (ER, PgR, HER2) was analyzed in pairs of BC and BCLM from each individual (Fig. 3). ER expression changed in 22/130 (16.9%) and among these, from ER positive to negative in 16/22 (72.7%). The ER positive BC converted in 16/93 (17.2%) and the ER negative BC in 6/37 (16.2%). Among the receptors, PgR converted most frequently, in 37/114 (32.5%) and most changed from positive to negative (32 /37, 86.5%). In the PgR positive primaries, a majority converted to a PgR negative phenotype in the BCLM (32/52, 61.5%) while only 5/62 (8.0%) of the PgR negative BC changed expression pattern in the BCLM. This resulted in a significantly increased ratio of PgR negative tumors in the liver 89/114 (78.1%) compared to the breast 62/114 (54.4%), (*p* < 0.001). For HER2, 10/101 tumors (9.9%) converted. Most HER2 conversions were from HER2-negative to the HER2-amplified phenotype (8/10, 80%). The HER2 conversion frequency in HER2-amplified and HER2-negative BC was 2/16 (12.5%) and 8/85 (9.4%) respectively).

### Subtype conversion

**Fig. 4:**
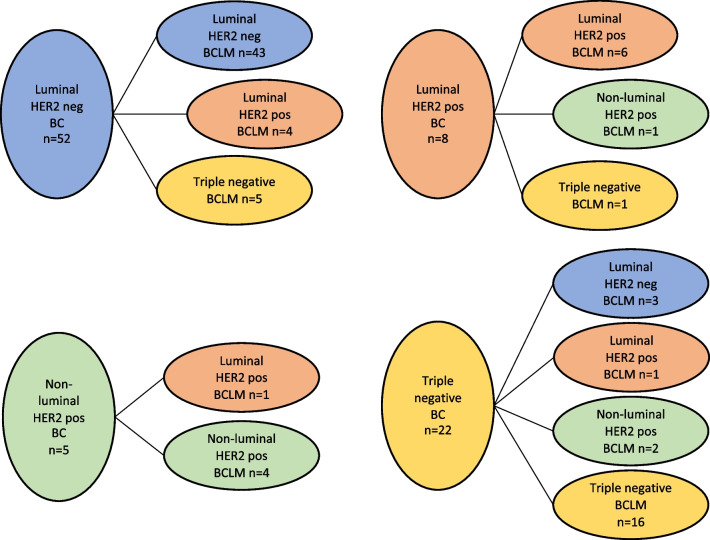
Subtype conversion from primary breast cancer (BC) to Breast Cancer Liver Metastases (BCLM). HER2, Human Epidermal growth factor receptor 2

Complete data on receptor expression in both BC and BCLM was available in 87 patients and these tumors were classified into subtypes as described (Fig. 2). Subtype conversion was seen in 18/87 patients (20.7%) (Fig. 4). If luminal and HER2 negative subtypes are considered prognostically favorable, most tumors converted to a subtype with a worse prognosis (11/18, 61.1%). However, among the remaining seven patients with a converted subtype, six had a triple negative primary tumor where the BCLM eventually expressed either luminal markers and/or HER2.

In the Luminal HER2 negative cohort, 43/52 (82.7%) remained unchanged, 4/52 converted to a Luminal HER2 positive BCLM and 5/52 (9.6%) to a triple negative tumor subtype. Conversion between Luminal A and Luminal B HER2 negative was not possible to evaluate due to lack of data for histologic grade and Ki-67. In the HER2 positive subtypes (luminal and non-luminal), 1/13 had a triple negative BCLM. In addition, one gained and one lost luminal expression, respectively.

### Predictive value and survival

Patients who had surgical treatment for BCLM (*n* = 15) were excluded from all survival analyses. In the remaining cohort of medically treated patients with BCLM (*n* = 117), median overall survival from time of BC diagnosis and time of BCLM was 46 months (95% CI 39.5–52.5) and 13 months (95% CI 8.6–17.4), respectively. To investigate predictive factors at time of BCLM diagnosis, a Cox regression model was used with time from BCLM to death or end of follow-up as outcome (Table 2). In the univariate analysis, age, NHG, multiple BCLM, ER or PgR negative primary tumors and PgR negative BCLM were associated with a poor survival. In addition, HER2 amplification in the primary tumor as well as in BCLM was a positive predictive factor. All factors with a *p* value < 0.2 in the univariate analysis were included in a multivariable analysis. Here, both PgR positivity and HER2 amplification in the primary BC had a significant positive impact on survival (HR 0.44 and 0.28 respectively) (Table 2).

**Table 2:** Predictive factors (Cox regression analysis)

	Univariate analysis	Multivariable analysis
Predictive factor	HR (95% CI); *p* value	HR (95% CI); *p* value
Age at BC	1.018 (1.002–1.033); **0.028**	0.867 (0.728–1.032); 0.109
Age at BCLM	1.017 (1.001–1.034); **0.036**	1.167 (0.980–1.390); 0.083
Time BC to BCLM	0.998 (0.990–1.007); 0.729	
TNM BC		
1	Ref = 1.000; 0.438	
2	0.739 (0.499–1.217); 0.235	
3	0.911 (0.455–1.825); 0.739	
NHG BC		
1	0.629 (0.228–1.738); 0.371	0.409 (0.112–1.485); 0.174
2	0.490 (0.315–0.764); 0.002	0.504 (0.275–0.924); 0.027
3	**Ref = 1.000; 0.007**	Ref = 1.000; 0.083
No of BCLM		
> 1	Ref = 1.000	Ref = 1.000
Single	**0.538 (0.322–0.896); 0.017**	0.545 (0.283–1.048); 0.069
Extrahepatic met		
Yes	Ref = 1.000	
No	1.007 (0.679–1.495); 0.971	
Conversion		
Yes	Ref = 1.000	
No	0.906 (0.552–1.485); 0.695	
Vascular invasion BC		
No	Ref = 1.000	
Yes	1.020 (0.641–1.623); 0.935	
ER BC		
Negative	Ref = 1.000	Ref = 1.000
Positive	**0.651 (0.427–0.994); 0.047**	0.711 (0.365–1.383); 0.315
PgR BC		
Negative	Ref = 1.000	Ref = 1.00
Positive	**0.588 (0.392–0.883); 0.010**	**0.443 (0.223–0.879); 0.020**
HER2 BC		
Negative	Ref = 1.000	Ref = 1.000
Positive	**0.471 (0.241–0.921); 0.028**	**0.277 (0.085–0.901); 0.033**
ER BCLM		
Negative	Ref = 1.000	
Positive	0.766 (0.505–1.162); 0.210	
PgR BCLM		
Negative	Ref = 1.000	Ref = 1.000
Positive	**0.468 (0.267–0.823); 0.008**	0.757 (0.341–1.682); 0.495
HER2 BCLM		
Negative	Ref = 1.000	Ref = 1.000
Positive	**0.604 (0.345–1.059); 0.078**	0.731 (0.292–1.828); 0.503

Covariates with *p* values < 0.2 in the univariate analysis are indicated in bold and were used in the multivariable analysis. *P* values < 0.05 in the multivariable analysis are indicated in bold. ER, Estrogen receptor; PgR, Progesterone receptor; HER2, Human Epidermal growth factor receptor 2; BC, primary breast cancer; BCLM, Breast Cancer Liver Metastases

In order to analyze if the receptor expression in the BC or the BCLM had the strongest predictive value, three different multivariable regression models were calculated. The first included only information about the receptor expression in the primary tumor while the second included BCLM status only. In the third analysis, receptor expression in both BC and BCLM was used. In addition to receptor status, the models included the covariates age at BC, NHG and number of BCLM since the univariate analysis indicates a possible impact on survival (*p* < 0.2 in Table 2). For these models, Harrell´s C-index was calculated. A C-index of 0.5 implies that the model is no better than chance and a higher index indicates that it is a better predictor of risk. The calculated C-indexes were 0.70 for only BC receptors, 0.68 for only BCLM receptors and 0.72 for both BC and BCLM status. Thus, the highest C-index was obtained when information from both BC and BCLM were used in the analysis. Very similar results were obtained when the same analysis was performed to analyze the predictive values of the subtypes in BC, BCLM and both combined (0.69, 0.69 and 0.70, respectively).

**Fig. 5:**
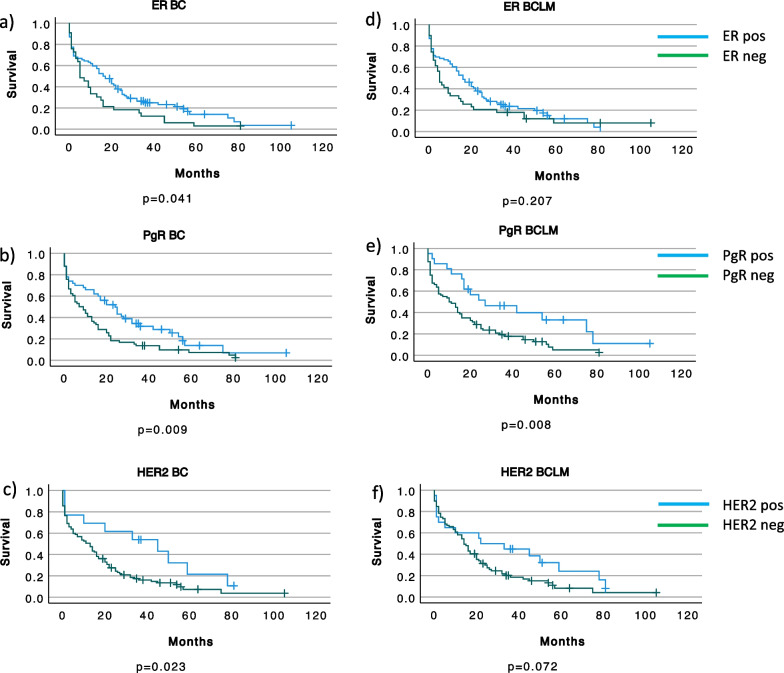
Survival from time of BCLM diagnosis in relation to receptor expressions in primary breast cancer (BC) (**a**–**c**) and Breast Cancer Liver Metastases (BCLM) (**d**–**f**). ER, Estrogen receptor; PgR, Progesterone receptor; HER2, Human Epidermal growth factor receptor 2

**Fig. 6:**
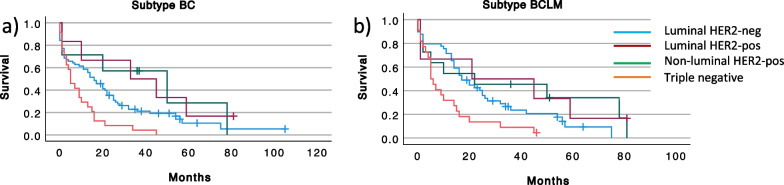
Survival from time of BCLM diagnosis in each BC subtype (**a**) and BCLM subtype (**b**). Luminal HER2-negative (blue), luminal HER2-positive (red), non-luminal HER2-positive (green) and triple negative (orange). BC, primary breast cancer; BCLM, Breast Cancer Liver Metastases

Kaplan–Meier plots and log rank tests were used to analyze survival related to receptor expression in BC and BCLM. As expected, BC expression of ER or PgR (Fig. 5a–b) was associated with improved survival (*p* = 0.041 and *p* = 0.009, respectively). PgR positive BCLM also had a favorable prognosis (*p* = 0.008) while ER positive BCLM failed to reach a significant advantage (Fig. 5d-e). Interestingly, Her2 amplified tumors were associated with improved prognosis (Fig. 5c and f). Finally, we analyzed survival after BCLM diagnosis related to the subtype (Fig. 2) of the BC (Fig. 6a) and the BCLM (Fig. 6b). As expected, triple negative tumors had the worst outcome. Both luminal and non-luminal HER2 positive tumors trended toward a better survival compared to the luminal HER2 negative subtype.

We conclude that receptor expression and subtype frequently change from BC to BCLM and that a liver biopsy is warranted to tailor treatment of BCLM. HER2 amplification is common in BCLM and constitutes a positive predictive factor in the era of HER2 directed treatment.

## Discussion

Receptor and subtype conversion occur frequently in breast cancer during the course of the disease. Here, we focused on liver metastases and found that the rate of receptor conversion in our cohort was similar to other studies [11]. In BCLM, most tumor conversions are the result of loss of ER/PgR expression or amplification of Her2. The Luminal A and B HER2 negative subtypes were merged for the purpose of this study. Thus, the total conversion rate was probably underestimated since conversions between these two subtypes were not detected.

In general, luminal HER2 negative tumors are expected to have a good prognosis in comparison with HER2 positive subtypes [17]. In our cohort, both luminal HER2 positive tumors and non-luminal HER2 positive tumors had a survival that seemed to exceed luminal HER2 negative tumors. One probable explanation for this is the effective targeted HER2 treatment. Other studies have shown that HER2 positive tumors have affinity for the liver as opposed to other metastatic sites [18]. In a study by Howlader et al., luminal HER2 positive tumors had a better survival than luminal HER2 negative tumors in stage IV breast cancer [17]. Also, the relative effect of tumor stage was greater than that of the subtype. Since all patients in our study had stage IV disease the effect of anti-HER2 treatment may be enhanced.

Importantly, one in four of the triple negative primary tumors converted to another subtype in the liver, all with other treatment options available. Overall, the triple negative cancers still have a dismal prognosis.

In our material, the predictive value for receptors and subtypes in BC and BCLM seem to be of equal importance. The predictive C-index increase when adding information from both BC and BCLM, although the increase was modest. In this material, we lack information on individual therapy after BCLM biopsy and cannot rule out the possibility that the BCLM profile had minor impact on treatment.

Since the subtype frequently change, our interpretation is that information about both the primary tumor and metastases is of importance to tailor the best treatment for each patient. Hence, a biopsy from BCLM is motivated despite risks of tumor cell seeding or bleeding. The frequency of seeding from BCLM may be less frequent, compared to colorectal liver metastases [14].

Oligometastases in the liver might be amenable to local treatment (surgery, ablation or radiotherapy) and this is currently investigated in a prospective trial [4]. In this scenario, tumor cell seeding is a serious issue. However, since we show that the receptor status converts in a third of the BCLM, we argue that the personalized targeted systemic treatment is of greater importance in the metastatic setting when compared to a potential risk of tumor cell seeding. Sampling of metastases is also advised in the latest guidelines for treatment of advanced breast cancer [1].

A strength of this study is that we focus on BCLM and the patients are recruited from registers with national coverage. To our knowledge, this is the largest study of receptor conversion in BCLM. In addition, the patients are identified during recent years when modern breast cancer treatment, including anti HER2 treatment was available. One limitation is that information about receptor expression was missing in some patients. This could result in an overestimation of survival times since missing data has been proven to be a negative predictive factor and generally more common in older patients, later stage disease and low socioeconomic status [17]. Another weakness is that we lack data on systemic treatment of the BCLM, although our analysis indicates that adjuvant treatment for the primary breast cancer was administered according to current Swedish national guidelines. As a consequence, we cannot draw any conclusions about how systemic treatment affects receptor conversion in this study. Other factors that might influence the rate and type of conversion are tumor heterogeneity, differences in sampling, spontaneous mutations and metastasis location [11].

## Conclusions

Despite the progress in breast cancer treatment, the results from this study shows that BCLM patients still have a poor prognosis. We conclude that ER, PgR and HER2 status frequently convert from BC to BCLM, leading to a novel subtype classification in 21% of the liver metastases. Thus, a liver biopsy is warranted to optimize treatment. HER2 positivity is a positive prognostic factor in patients with BCLM.

## Data Availability

Supporting data are available by request to the corresponding author in accordance with regulations from NCBR and SweLiv.
